# Visual appearance interacts with conceptual knowledge in object recognition

**DOI:** 10.3389/fpsyg.2014.00793

**Published:** 2014-07-29

**Authors:** Olivia S. Cheung, Isabel Gauthier

**Affiliations:** ^1^Department of Psychology, Harvard UniversityCambridge, MA, USA; ^2^Center for Mind/Brain Sciences, University of TrentoTrentino, Italy; ^3^Department of Psychology, Vanderbilt UniversityNashville, TN, USA

**Keywords:** object learning, semantics, visual features, perceptual expertise

## Abstract

Objects contain rich visual and conceptual information, but do these two types of information interact? Here, we examine whether visual and conceptual information interact when observers see novel objects for the first time. We then address how this interaction influences the acquisition of perceptual expertise. We used two types of novel objects (Greebles), designed to resemble either animals or tools, and two lists of words, which described non-visual attributes of people or man-made objects. Participants first judged if a word was more suitable for describing people or objects while ignoring a task-irrelevant image, and showed faster responses if the words and the unfamiliar objects were congruent in terms of animacy (e.g., animal-like objects with words that described human). Participants then learned to associate objects and words that were either congruent or not in animacy, before receiving expertise training to rapidly individuate the objects. Congruent pairing of visual and conceptual information facilitated observers' ability to become a perceptual expert, as revealed in a matching task that required visual identification at the basic or subordinate levels. Taken together, these findings show that visual and conceptual information interact at multiple levels in object recognition.

## Introduction

A chocolate bunny is more visually similar to a stuffed animal but more conceptually similar to a baking chocolate bar, and the combination is such that a child may not allow her parent to melt it to bake a cake, nor would the parent allow the child to bring it in bed. Our interactions with objects must take both visual and conceptual information into account but little research addresses how object recognition mechanisms are constrained by the *interactions* between these two sources of information.

Object perception involves more than processing visual features. For familiar objects, visual knowledge, such as color of a fruit, modulates perception of salient features of an object (Hansen et al., 2006; Witzel et al., 2011). Conceptual knowledge about familiar object categories is also represented in the visual system (e.g., animals, tools, Chao et al., 1999; Mahon and Caramazza, 2009; Huth et al., 2012). While is often assumed that visual features of novel objects engage minimal conceptual processing (Tarr and Pinker, 1989; Bülthoff and Edelman, 1992; Gauthier and Tarr, 1997; Hayward and Williams, 2000; Schwoebel and Srinivas, 2000; Curby et al., 2004; Bar and Neta, 2006; Op de Beeck et al., 2006), shape dimensions of novel objects (e.g., sharpness, symmetry, contrast, complexity) can impact observers' subjective preferences (Reber et al., 2004; Bar and Neta, 2006, 2007). Moreover, intuitions may also be formulated about the similarity of novel objects to familiar objects (e.g., smooth novel objects resembling “women wearing hats,” Op de Beeck et al., 2006, p.13031), and such meaningful interpretations of ambiguous shapes appear to be robust and stable within individual observers (Voss et al., 2012). However, how meanings evoked by visual features may influence object processing remains a question that has not been explored systematically.

Some information on how object representations are constrained by both visual and conceptual factors comes from experiments where new conceptual associations are created for visual stimuli. Conceptual associations can facilitate perceptual categorization (Wisniewski and Medin, 1994; Lin and Murphy, 1997), bias perceptual interpretation of neutral stimuli (Bentin and Golland, 2002; Hillar and Kemp, 2008), and improve visual discrimination (Dux and Coltheart, 2005; Lupyan and Spivey, 2008). The discriminability of shapes or faces increases after having been paired with words from different categories, compare with having been paired with words from similar categories (Dixon et al., 1997, 1998; Gauthier et al., 2003). Observers also activate recent conceptual associations during visual judgments, even when the information is task irrelevant (James and Gauthier, 2003, 2004). However, in these studies (e.g., Dixon et al., 1997, 1998; Gauthier et al., 2003; James and Gauthier, 2003, 2004), the conceptual and visual information are arbitrarily associated, leaving entirely open whether some of these associations are created more easily than others, such as when the visual and conceptual features convey congruent, compared to contradictory, information.

We start with the assumption that the animate/inanimate distinction exists in the visual arena (objects can look like an animal or not) as well as in the non-visual conceptual arena (we can list attributes of objects that are animate or not). In this study, we manipulated both visual and conceptual features to study their interaction, more specifically the alignment of an animate vs. inanimate dimension in the visual and conceptual domains. We used words that described non-visual attributes that would normally apply to either people or man-made objects (e.g., cheerful, affordable), and created novel objects that resembled either living or non-living things. For visual features, we attempted to convey the animate vs. inanimate character of novel objects by manipulating shape, texture and color. These dimensions were chosen because bilateral shape symmetry is a powerful indicator of animacy (Concar, 1995; Rhodes et al., 1998), whereas the shape of man-made objects is more variable depending on their function. Also, the objects were rendered in colors and textures generally associated with animals or tools (e.g., skin color/organic vs. non-skin color/metallic). Experimental manipulation of both conceptual and visual information afforded us more control to investigate their interaction.

### Initial visual-conceptual biases

We first examined to what extent the visual appearance of novel objects from unfamiliar categories evokes conceptual processing, when observers see the objects for the first time. We asked whether visual features of the “animal-like” and “tool-like” object sets are sufficient to evoke the conceptual biases of animacy. Instead of asking participants directly to categorize the novel objects as animate or inanimate entities, we tested if the visual appearance of the objects evoked the concepts related to animate vs. inanimate categories by testing whether their (task-irrelevant) presence interfered with judgments of non-visual attributes as being more relevant to people or to man-made objects (e.g., “excited,” “grateful” vs. “durable,” “useful”).

### Visual-conceptual interaction on expert recognition

Beyond any early conceptual biases evoked by visual appearance, it is also possible that visual-conceptual interactions become more important with experience with a category. If visual features of novel objects activate abstract biases, anchoring the objects into existing conceptual networks appropriately (e.g., calling animate-like objects “animals” vs. calling tool-like objects “animals”) may constrain their representations during expertise training. There may be differences in the acquisition of expertise between objects that look like animals or not (i.e., the effect of visual appearance), or between objects that are introduced as having animate or inanimate conceptual properties (i.e., the effect of conceptual associations). But more importantly, we asked whether it is easier to acquire expertise with a category that is assigned conceptual features congruent with its appearance (i.e., the interaction between visual and conceptual information), as we conjectured that learning objects with congruent visual and conceptual information might enhance the ability to locate diagnostic visual features for fine-level discrimination.

### Training procedures

Here we combined training procedures used in previous conceptual association studies (James and Gauthier, 2003, 2004) and expertise studies (Gauthier and Tarr, 1997, 2002; Wong et al., 2009). During the two-stage training, participants first learned to associate particular concepts with individual objects, and then learned to rapidly recognize objects at the subordinate level. Critically, participants were divided into two groups during the first training stage: Both groups were shown identical words and objects, but the Congruent pairing group learned to associate animate attributes with animal-like objects and inanimate attributes with tool-like objects, while the Incongruent pairing group learned the opposite pairings. In the second training stage, both groups practiced individuating objects from both animal-like and tool-like categories, without further mention of conceptual information.

### Dependent measures

We used two dependent measures to reveal potential visual-conceptual interactions. First, in a word judgment task, participants categorized words as appropriate for describing people or man-made objects presented on task-irrelevant objects. This task was first completed prior to any training, and then completed after each training stage. This task uses an opposition logic similar to the Stroop task (1935) and several tasks since (e.g., see Bub et al., 2008), to test whether the visual appearance of the animal-like and tool-like objects would be sufficient to evoke concepts relevant to animacy/non-animacy. If our manipulation of visual appearance does not evoke animate vs. inanimate concepts, word judgment performance should not be affected by whether congruent or incongruent objects are present. While the actual locus of any interference may be at the response level, such responses would have to be evoked by visual appearance (note that at pre-test, no response had ever been associated with these or similar objects).

Second, in an object matching task, participants judged if two objects were from the same category (basic-level trials), or showed the same individual (subordinate-level trials). The reduction of the “basic-level advantage” is a hallmark of real-world expertise (Tanaka and Taylor, 1991), which is also sensitive to short-term expertise training (Bukach et al., 2012). Expert observers recognize individual objects in their expert categories at the *subordinate level* (e.g., “eastern screech owl,” or “Tom Hanks”) as quickly as at the *basic level* (e.g., “bird,” or “man”), whereas novices recognize the objects faster at the basic than the subordinate levels (i.e., the “basic-level advantage,” Rosch et al., 1976). The basic-level advantage is reduced in experts for both animate and inanimate object categories (e.g., faces: Tanaka, 2001; birds: Tanaka et al., 2005; Scott et al., 2006, 2008; novel 3D objects: Gauthier et al., 1998; Wong et al., 2009; Wong et al., 2012). With novel objects, explicit conceptual information is often absent during training (e.g., Wong et al., 2009; Wong et al., 2012). Although faster subordinate-level processing in experts might depend predominantly on experience with perceptual information of similar exemplars in a category, it is possible that conceptual information also impose processing constraints. For instance, brief learning of a diverse set of semantic associations with novel objects can facilitate subordinate-level judgment compared to that of a restricted set (Gauthier et al., 2003). The question of interest here is whether observers apply conceptual knowledge about familiar categories to novel objects, based on the visual resemblance between the familiar and novel categories. If this is the case, conceptual information expected based on past experience with visual features may facilitate fine-level discrimination of similar exemplars, as both visual and conceptual information interact to constrain object representations.

Here, we assessed whether having associated concepts that are congruent with the visual appearance of a category may facilitate the recognition of the objects at the subordinate-level compared to the basic-level, even though the object matching task can be accomplished based on visual features alone. We measured any differences in the “basic-level advantage” after semantic training and after individuation training. If visual processing is facilitated by visual-conceptual pairings, then the basic-level advantage should be more reduced in participants who associated the objects with congruent conceptual features than in those who received incongruent pairings.

## Methods

### Participants

Twenty-four adults (normal/corrected-to-normal vision) from Vanderbilt University participated for payment ($12/h). The study was approved by the Vanderbilt University IRB. Participants were randomly assigned to the Congruent pairing group (6 females and 6 males, age *M* = 22.58, *SD* = 4.32) or the Incongruent pairing group (4 females and 8 males, age *M* = 23.67, *SD* = 4.29). Twelve additional adults (5 females and 7 males, age *M* = 22.67, *SD* = 3.08) participated only in the object-matching task once as a Control group.

### Stimuli

#### Objects

**Figure 1:**
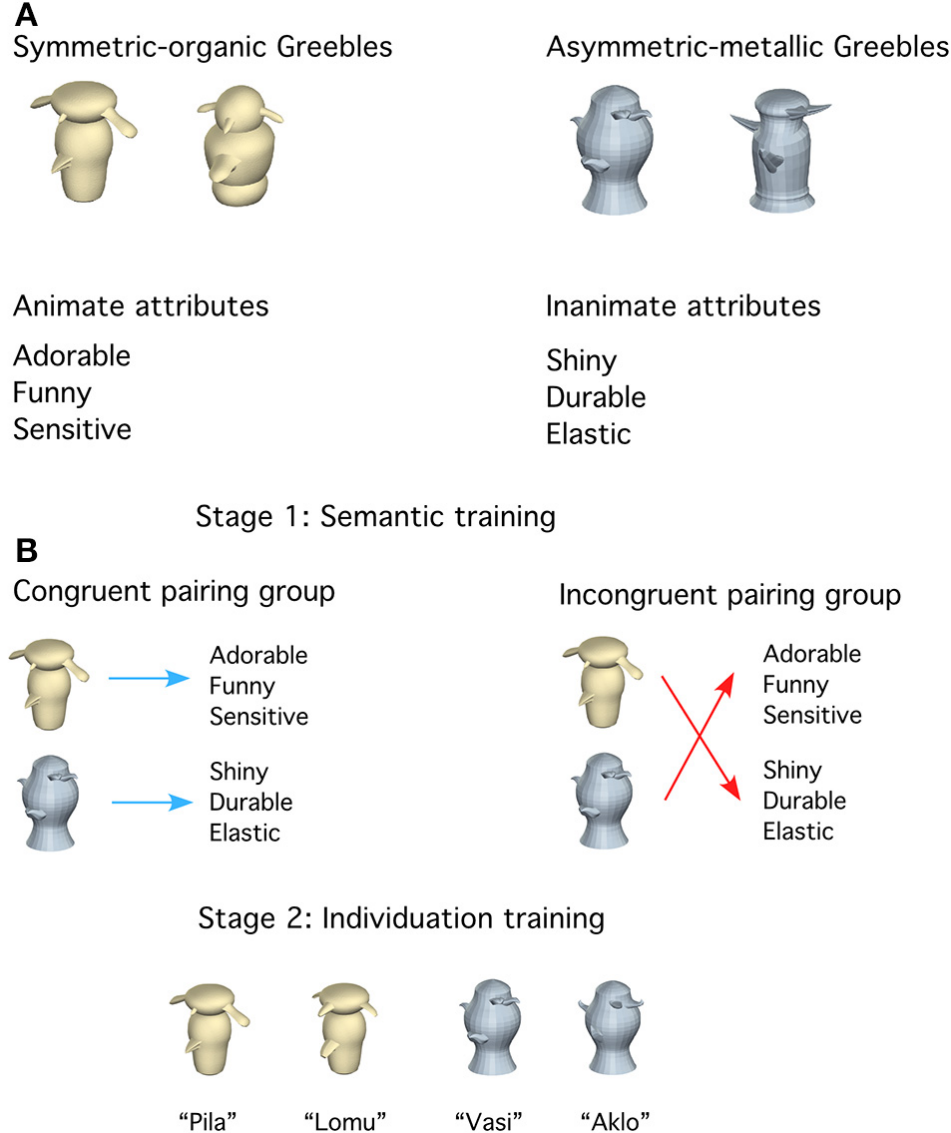
**(A)** Examples of the two categories of objects and two categories of words, including Symmetric-organic objects, Asymmetric-metallic objects, animate attributes and inanimate attributes. **(B)** Schematic of the two-stage training. In semantic training (stage 1), participants were divided into two groups to learn to associate three words to each trained object. The two training groups differed only in terms of the *pairing* of the objects and words. In individuation training (stage 2), all participants learned to name and identify objects at the subordinate level quickly and accurately.

Each participant was shown 48 novel objects called “Greebles” (see examples in Figure 1A) created using 3D Studio Max. Half of the objects (24) were *Symmetric-organic Greebles* with smooth-edged parts and organic textures. The rest (24) were *Asymmetric-metallic Greebles* with sharp-edge parts and metallic textures. Note that symmetry refers to object, and not image, symmetry. Each Greeble had a unique set of four peripheral parts. To minimize object-specific effects, we generated two versions of Symmetric-organic and Asymmetric-metallic Greebles that differed in color (i.e., yellow/pink, blue/green), central and peripheral part assignment to the objects. Each version was shown to half of the participants in each of the two training groups. There were 18 Greebles from each of the Symmetric-organic and Asymmetric-metallic categories in the trained subsets, and 6 in the untrained subsets (which were used as foils in the basic-level recognition task). The two subsets (trained or untrained) within each category had different central and peripheral parts. From each trained subset, six Greebles were used in semantic training. An additional six Greebles from each trained subset were also used in individuation training. All objects were shown during the testing tasks. The objects used for training and testing were counterbalanced across participants within each group and matched between groups. All Greebles were rendered on a white background at four viewpoints (0/6/12/18°: The 0° view was an arbitrarily defined orientation with the symmetric axis rotated 40° to the right). The image size was approximately 6 × 3.6° of visual angle. To avoid image-based effects, objects used during training were shown at 0 and 18°. During testing, the objects were presented at 6 and 12°. Additionally, phase-scrambled images of the Greebles were also created as control stimuli in one of the tasks.

#### Words

Eighty-four words were used; each described a *non-visual* attribute appropriate for describing either people (“animate attributes”) or man-made objects (“inanimate attributes”; Figure 1A and Appendix A in Supplementary Material), generated in a pilot study (*N* = 20). Word length was controlled across the animate (*M* = 7.17 letters, *SD* = 2.05) vs. inanimate (*M* = 7.5 letters, *SD* = 1.86) features. According to the SUBTLEX_us_ word frequency database (Brysbaert and New, 2009), the mean frequency was higher for the animate (*M* = 38.06, *SD* = 63.53) than inanimate (*M* = 4.16, *SD* = 6.29) attributes. But since the critical manipulation here was the object-word *pairing* and identical words were used for both training groups, word frequency alone could not account for differences between groups. Twenty-four animate and 24 inanimate attributes were used in the word judgment task. Eighteen animate and 18 inanimate attributes were used during semantic training. The words used were also counterbalanced across participants within each group and matched between groups.

### Procedure

The study was conducted on Mac mini computers with 19″ CRT monitors using Matlab. Below are the details on the two-stage training (which lasted approximately 9 h across 6 days), the word judgment and object matching tasks (which lasted 15 and 45 min respectively). The entire study consisted of a pre-test (word judgment task), followed by two sessions of semantic training, followed by a session with two post-test tasks (word judgment and object matching), followed by four sessions of individuation training, then another session with the two post-test tasks.

#### Training

***Training stage 1: semantic training***. During semantic training (two 90-min sessions; Figure 1B, Table 1), each group learned three randomly selected words each for 12 Greebles (6 Symmetric-organic Greebles and 6 Asymmetric-metallic Greebles). The Congruent pairing group learned animate attributes with Symmetric-organic Greebles and inanimate attributes with Asymmetric-metallic Greebles, whereas the Incongruent pairing group learned the opposite pairing. Identical sets of word triplets were assigned to one participant in the Congruent pairing group and another in the Incongruent pairing group. The two categories of Greebles were shown in interleaved blocks.

**Table 1:** **Task details of the two-stage training paradigms**.

**SEMANTIC TRAINING**		
**Session and number of objects involved**	**No. of trials**	**Task**
Session 1 (4 Symmetric-organic Greebles and 4 Asymmetric-metallic Greebles),	16 in session 1,	Passive viewing: To initiate learning, this task allowed participants to study each Greeble with the three associated attributes, twice for as long as needed
Session 2 (6 Symmetric-organic Greebles and 6 Asymmetric-metallic Greebles)	24 in session 2	
	576	Three-attribute matching: To promote associations between each Greeble and each unique set of attributes, this task required participants to judge if a set of three attributes matched a concurrently presented Greeble
	576	Single-attribute matching: To ensure participants learned all three attributes independently, instead of any one from each set, this task required participants to judge if a single attribute matched a subsequently presented Greeble
	16 in session 1,	Recall: To examine if participants were able to generate the associated attributes without verbal hints, participants were asked to input the three attributes associated with each Greeble, twice
	24 in session 2	
**INDIVIDUATION TRAINING**		
**Session and number of objects involved**	**No. of trials**	**Task**
Session 1 (6 Symmetric-organic Greebles and 6 Asymmetric-metallic Greebles),	720	Naming: To promote learning of each Greeble with its name, participants were asked to input the first letter of the name associated with a Greeble. The names were shown during the first 3 presentations of a Greeble
Session 2 (12 Symmetric-organic Greebles and 12 Asymmetric-metallic Greebles)		
	480	Name matching: To ensure participants learn to individuate the Greebles quickly and accurately, participants were asked to judge if a name matched with a concurrently presented Greeble as quickly and accurately as possible
	384	Name verification: A variation of the Name matching task to encourage task-general learning, participants judged if a name matched with a subsequently presented Greeble

*We used a variety of tasks in every session to promote task-general learning. These tasks were previously used in several studies (semantic training: James and Gauthier, 2003, 2004; individuation training: e.g., Gauthier and Tarr, 2002; Wong et al., 2009). The semantic training (stage 1) consisted of four tasks promoting associations between a set of three words to a trained object. The trained objects were introduced across the first two training sessions: 8 Greebles (4 Symmetric-organic and 4 Asymmetric-metallic Greebles) were introduced in session 1 and all 12 Greebles (6 of each category) were introduced in session 2. The individuation training (stage 2) consisted of three tasks that aimed to enhance the speed and accuracy of identification for individual objects at the subordinate level. The trained objects were introduced across the first two training sessions: 12 Greebles were used in session 1 and all 24 Greebles were used in sessions 2–4*.

***Training stage 2: individuation training***. During individuation training (four 90-min sessions; Figure 1B, Table 1), all participants learned to individuate 24 Greebles (12 Symmetric-organic and 12 Asymmetric-metallic Greebles; in which 6 from each category were previously shown during semantic training). Additional objects were used in this phase to increase the difficulty of rapid identification. During this training, each Greeble was named with a 2-syllable nonsense word (e.g., Pila, Aklo, see Appendix B in Supplementary Material for the full list). Name assignment was randomized within group but matched between groups. Both speed and accuracy were emphasized in all training tasks. To motivate participants, the mean speed and accuracy for each block were shown at the end of each block. Symmetric-organic and Asymmetric-metallic Greebles were shown in interleaved blocks.

#### Testing

**Figure 2:**
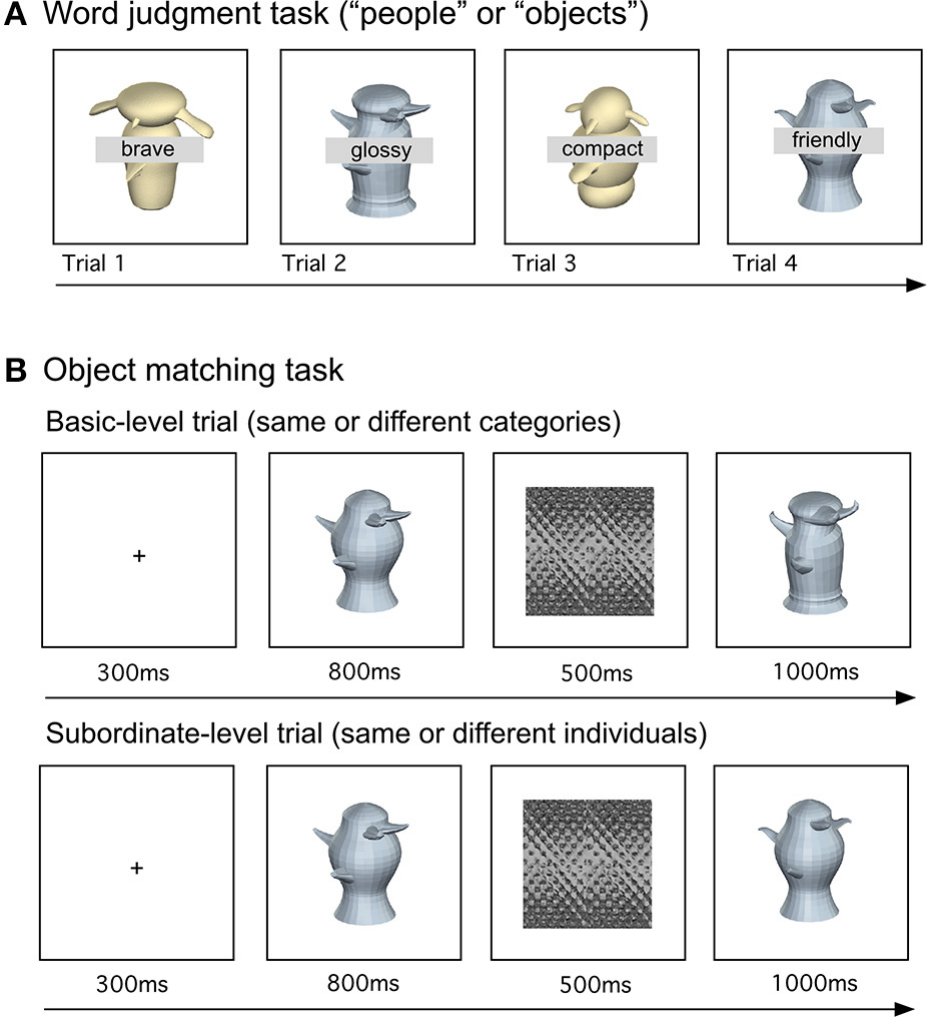
**Example trials of (A) the word judgment task and (B) the object matching task at the basic level (top: a basic-level trial with two individuals from different categories) and at the subordinate level (bottom: a subordinate-level trial with two individuals from the same category)**.

***Word judgment***. Participants first completed the 15-min task (Figure 2A) prior to training, and again after semantic training and after individuation training. In this 2-alternative forced choice task, participants judged if a word was more appropriate for describing people or objects, while told to ignore an image presented behind each word. In a total of 432 trials, each of the 24 animate and 24 inanimate attributes was presented nine times. Each word appeared twice with each of the 48 Symmetric-organic Greebles and 48 Asymmetric-metallic Greebles at each of two slightly different viewpoints (difference = 6°), and four times with each of 12 phase-scrambled Greeble images (6 Symmetric-organic and 6 Asymmetric-metallic). The phase-scrambled images were included to evaluate whether participants paid additional attention to the task-irrelevant Greebles during the word judgment task. All stimuli were shown until a response, with a 1-s interval in between trials. All conditions were randomized.

#### Matching at basic- and subordinate-levels

Participants completed this 45-min task (768 trials) after semantic training and after individuation training (Figure 2B). In different blocks, participants judged if two sequentially presented objects were identical or different, at either the basic or subordinate level. In basic-level blocks, object pairs could be Greebles from the same category (the same central body part) or different categories (different central body parts). In subordinate-level blocks, the object pairs could be identical or different individuals from the same category (the same central body parts but different peripheral parts). All object pairs were shown across 6° rotation. The following conditions were blocked: Categorization level (basic/subordinate), Visual appearance (Symmetric/Asymmetric), and training status (trained/untrained objects). On each trial, a 300 ms-fixation was followed by a study image (800 ms), a mask (500 ms), and by a test image (1 s).

## Results

### Training results

**Figure 3:**
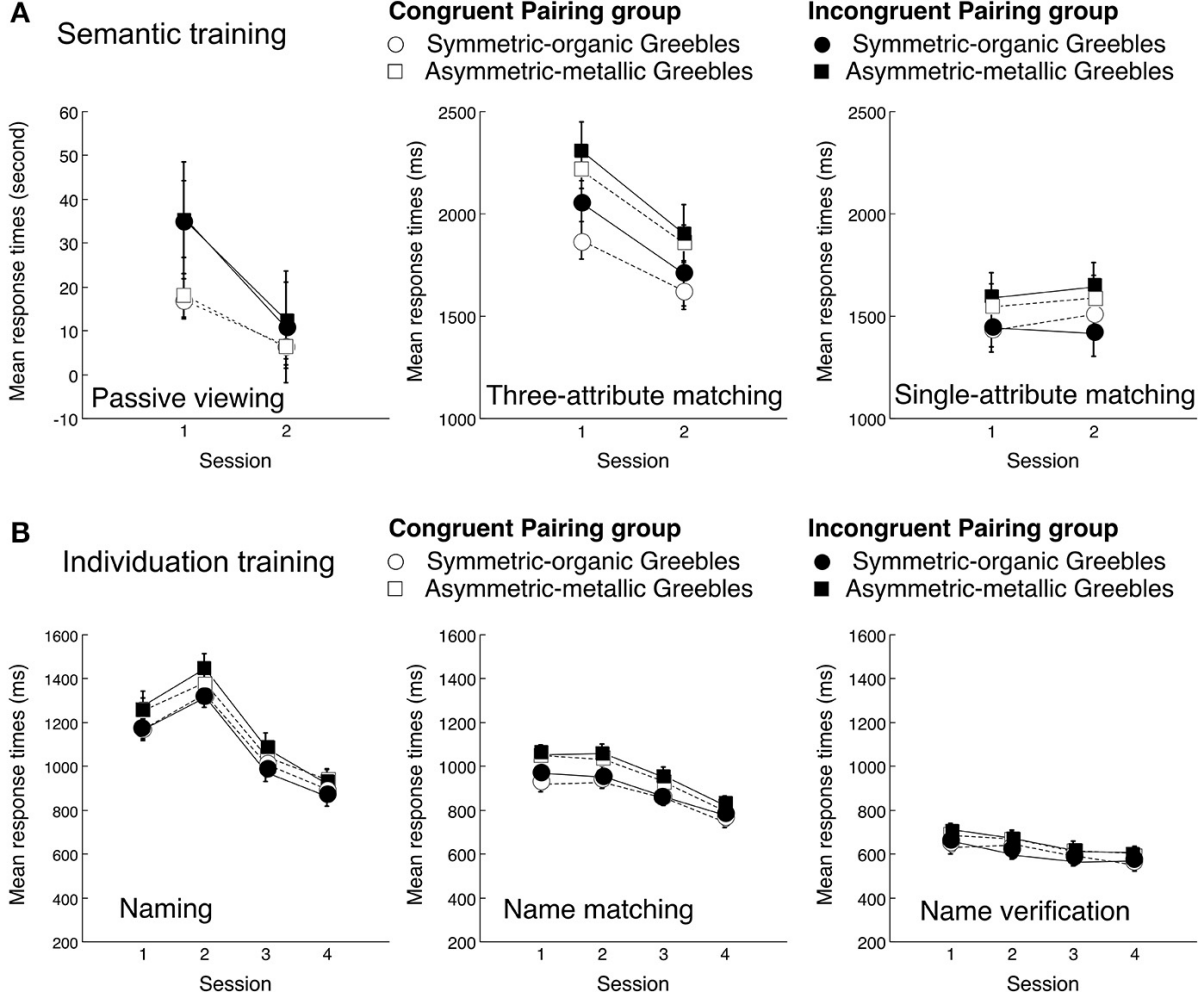
**Mean response times in the various tasks of (A) semantic training and (B) individuation training**. Note that RT was in seconds in the Passive viewing task and in milliseconds in all other tasks. Error bars represent the 95% confidence intervals of the training effects across sessions within each group for each object type.

The training was meant to form conceptual associations and improve individuation performance, and the training results (Figure 3) were not a focus of the study. Both groups showed accuracy near ceiling throughout training (i.e., well above 90% in all tasks across all sessions), with the expected significant increases in all individuation training tasks. Responses became faster with time in all semantic training and individual training tasks but the single-attribute matching task. Note that responses were also faster for Symmetric-organic Greebles than Asymmetric-metallic Greebles, but there was no statistical significant difference in performance between the groups in all but the passive viewing task during semantic training. We do not report statistical analyses here, but Figure 3 shows confidence intervals relevant to the significant training effects across sessions.

### Testing results

#### Word judgment

We focused on RT in correct trials because accuracy in this task was high (>95%)^1^.

**Figure 4:**
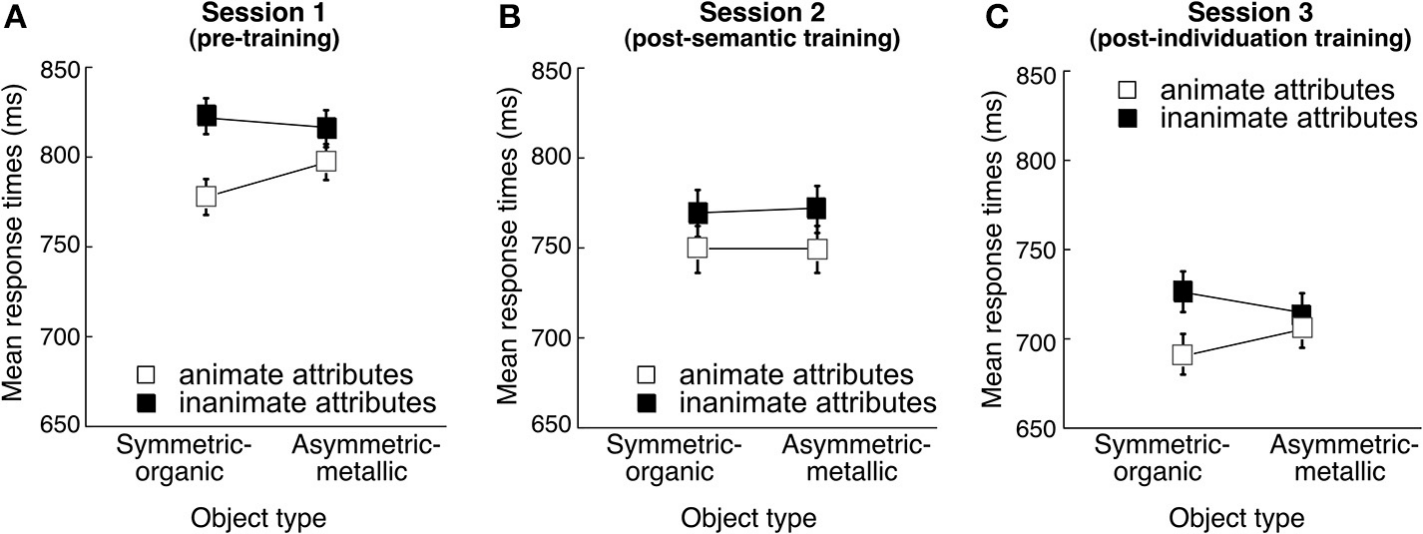
**Mean response times (ms) in the word judgment task as a function of Word type (animate attributes vs. inanimate attributes) and Object type (Symmetric-organic Greebles vs. Asymmetric-metallic Greebles) (A) in the first session (pre-training), (B) in the second session (post-semantic training), and (C) in the third session (post-individuation training)**. Error bars represent the 95% confidence intervals of the Word type and Object type interaction. The lines were added to the figures despite the conditions being categorical, to highlight the interactions.

There was no effect of the Pairing group on this task at any stage of the study (pre-test, after semantic training or individuation training) ANOVAs (all *p* > 0.35). There results are therefore presented in Figure 4 collapsing over this factor.

It was entirely expected that there would be no difference between the two pairing groups at pre-test because no pairings had actually been done. At this stage, the question was whether the visual appearance of novel objects imply conceptual information about animacy. We also measured performance in the word judgment in a baseline condition where task-irrelevant scrambled images were shown behind the words (Table 2).

**Table 2:** **Mean response times (ms) in the word judgment task for each image type (Symmetric-organic Greebles, Asymmetric-metallic Greebles, and scrambled images) across the three testing sessions**.

	**Symmetric-organic Greebles**	**Asymmetric-metallic Greebles**	**Scrambled images**
Session 1	798.9 (25.2)	805.7 (24.5)	830.9 (28.7)
Session 2	758.0 (29.0)	759.3 (28.4)	768.2 (27.3)
Session 3	707.5 (21.4)	709.5 (21.2)	723.2 (19.5)

*Standard errors of the mean are reported within the parentheses*.

As mentioned above, we observed no effect of Pairing groups after pairings were learned in semantic training. Like any null result, this is difficult to interpret, but given we found other effects of pairing group in the study (described later), this suggests that the word judgment is simply not sensitive to these effects. This could be because it is an explicit conceptual task in which participants can as easily retrieve all the explicitly learned associations, whether congruent or incongruent. In contrast, in a more perceptual task where no explicit conceptual search is activated, congruent visual-conceptual pairings may show more of an advantage.

***Interaction between visual appearance and conceptual information***. After collapsing over the non-significant factor of pairing group, we focus here on the effect of Visual appearance on word categorization across sessions (Figure 4). A Session (pre-training/post-semantic training/post-individuation training) × Word type (animate/inanimate) × Object type (Symmetric-organic/Asymmetric-metallic) ANOVA was conducted. Responses became faster with time, *F*_(2, 46)_ = 13.53, ${\tilde{\eta }}_{p}^{2}$ = 0.37, *p* < 0.0001. Responses were also faster for judging animate than inanimate attributes, *F*_(1, 23)_ = 10.60, ${\tilde{\eta }}_{p}^{2}$ = 0.32, *p* = 0.0035, possibly because of the higher word frequency for animate than inanimate attributes. There was no significant effect of Object type, *F*_(1, 23)_ = 0.86, ${\tilde{\eta }}_{p}^{2}$ = 0.04, *p* = 0.36, nor significant interactions between Session and Word type, *F*_(2, 46)_ = 0.48, ${\tilde{\eta }}_{p}^{2}$ = 0.02, *p* = 0.62, or between Session and Visual appearance, *F*_(2, 46)_ = 0.35, ${\tilde{\eta }}_{p}^{2}$ = 0.015, *p* = 0.71. Critically, however, Word type and Object type interacted, *F*_(1, 23)_ = 6.68, ${\tilde{\eta }}_{p}^{2}$ = 0.25, *p* = 0.017, although the 3-way interaction of Session, Word type and Object type did not reach significance, *F*_(2, 46)_ = 2.31, ${\tilde{\eta }}_{p}^{2}$ = 0.09, *p* = 0.11.

One of our main goals was to investigate whether Word type and Visual appearance might interact when participants were first presented with the objects during the pre-training sessions, and how a putative interaction would be affected by further training. Therefore, we conducted a Word type (animate/inanimate) × Object type (Symmetric-organic/Asymmetric-metallic) ANOVA separately for each session to examine if the effect was already significant at pre-test (Figures 4A–C). Critically, we found a significant interaction between Word type and Object type in pre-training [*F*_(1, 23)_ = 7.18, ${\tilde{\eta }}_{p}^{2}$ = 0.24, *p* = 0.013]. Scheffé's *post-hoc* tests revealed faster judgment for animate attributes with the presence of Symmetric-organic Greebles compared with Asymmetric-metallic Greebles (*p* = 0.0045), and a significant effect of the opposite result for judging inanimate attributes (*p* = 0.015).

The Word type and Object type interaction was also significant after individuation training [*F*_(1, 23)_ = 6.16, ${\tilde{\eta }}_{p}^{2}$ = 0.21, *p* = 0.02], but interestingly, it was not immediately after semantic training [*F*_(1, 23)_ = 0.033, ${\tilde{\eta }}_{p}^{2}$ = 0.0015, *p* = 0.86]. We observe a bias for relating novel animal-like (or tool-like) objects to human (or object) attributes at pre-test, and it seems that introducing explicit semantic associations can temporarily alter this bias. This is also consistent with the idea, suggested above to explain the lack of a Pairing group effect, that this explicit word judgment task may be most sensitive to implicit influences. During semantic training, participants in all groups had to learn associations with the objects, and the training ensured that all associations were learned. These explicit associations would have been more salient to the minds of participants in Session 2 than later on. We would therefore speculate that these associations blocked the effects of visual appearance that we observe in Sessions 1 and 3, and the reappearance of the interaction effect in Session 3 demonstrates that the faster RTs or practice with the word judgment task cannot account for the lack of effect in Session 2.

***Manipulation check: objects vs. scrambled objects as task-irrelevant images***. To test whether participants paid less attention to the words during the word judgment task due to the presence of task-irrelevant objects, we compared performance to that for the same task with words shown on scrambled images. The presence of an object was apparently not more distracting than the presence of a scrambled image, in fact if anything the objects were easier to ignore than the scrambled images (perhaps due to low-level image properties). Indeed, RTs for the word judgment were consistently faster when objects were present relative to scrambled images (Table 2). A Session (pre-training/post-semantic training/post-individuation training) × Image type (Symmetric-organic/Asymmetric-metallic/Scrambled) ANOVA showed an effect of Image type, *F*_(2, 46)_ = 8.26, ${\tilde{\eta }}_{p}^{2}$ = 0.26, *p* < 0.001, with faster RT with the presence of either type of objects compared to the scrambled images (*p*s < 0.01), and no difference between object types (*p* = 0.80). There was also an effect of Session, *F*_(2, 46)_ = 15.53, ${\tilde{\eta }}_{p}^{2}$ = 0.40, *p* < 0.0001, with faster RT as the sessions progressed, and no interaction between Session and Image type, *F*_(4, 92)_ = 1.11, ${\tilde{\eta }}_{p}^{2}$ = 0.05, *p* = 0.37.

#### Matching at the basic- and subordinate-levels

After finding that the visual appearance of novel objects can activate conceptual information in a word judgment task on the first encounter with these objects, we then examined the influence of acquired conceptual associations with animate vs. inanimate objects, in a matching task at the basic- and subordinate-levels. As in prior work (e.g., Gauthier and Tarr, 1997; Wong et al., 2009; Wong et al., 2011), we focus only on trials with unfamiliar objects from the trained categories that were not used during training (i.e., “transfer” objects)^2^, as a critical aspect of expertise is generalization of the skills to unfamiliar exemplars in the expert domain (e.g., car experts viewing cars, Bukach et al., 2010, face experts viewing faces, Tanaka, 2001). Here, we measured both response times (RT) and sensitivity (d′: z(hit rate)-z(false alarm rate)). We first compared the performance of the two training groups after semantic training to an untrained control group. We then compared the effects in the two training groups after both stages (semantic and individuation) of training.

**Figure 5:**
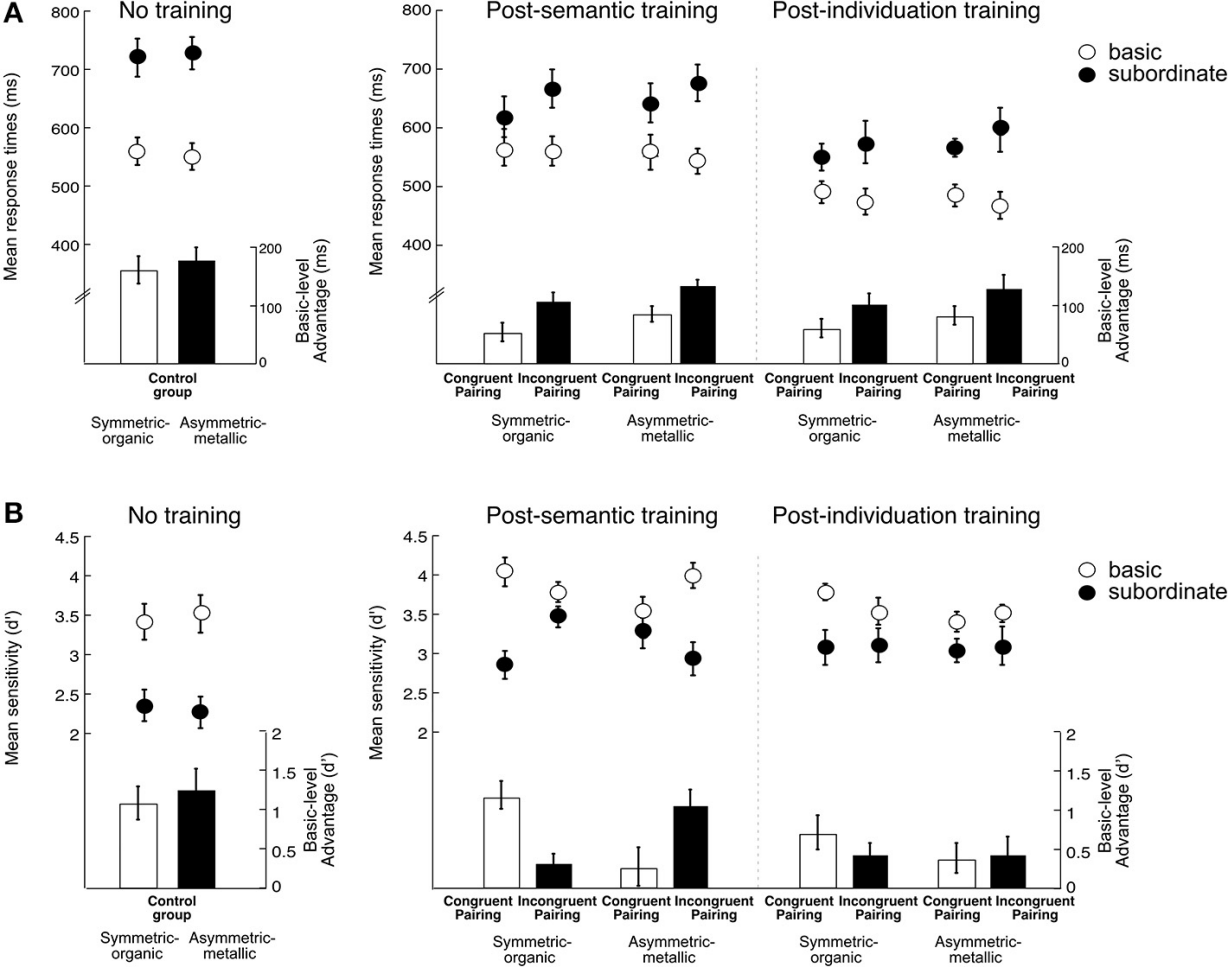
**Results of the object matching task**. Left panel: Performance of the Control group who did not receive any training. Right panel: Performance of the two Training groups. Panel **(A)** shows response times (ms) and Panel **(B)** shows sensitivity (d') as a function of Group/Pairing (Control vs. Congruent pairing vs. Incongruent pairing), Session (no training vs. post-semantic vs. post-individuation training), Visual appearance (Symmetric-organic Greebles vs. Asymmetric-metallic Greebles) and Categorization level (Basic vs. Subordinate). The dots represent mean response times or mean sensitivity, and the bars represent the mean basic-level advantage. Error bars represent the standard errors of the mean.

***Effects of semantic training (comparison between a Control group and the training groups)***. Semantic training with a few exemplars was sufficient to reduce basic-level advantage, even for untrained exemplars in the training groups compared to a Control group that did not receive any training (Figures 5A,B). A Group (Control/Congruent/Incongruent) × Object type (Symmetric-organic/Asymmetric-metallic) × Categorization level (Basic/Subordinate) ANOVA revealed a significant interaction of Group and Categorization level in RT, *F*_(1, 33)_ = 9.00, ${\tilde{\eta }}_{p}^{2}$ = 0.40, *p* < 0.001: the basic-level advantage was smaller in the training groups compared to the control group (*p*s < 0.05), and also smaller in the Congruent than Incongruent pairing group (*p* = 0.04). The Group and Categorization level interaction did not reach significance d', *F*_(1, 33)_ = 2.72, ${\tilde{\eta }}_{p}^{2}$ = 0.12, *p* = 0.08.

***Effects of both semantic and individuation training (comparison between the training groups)***. We then assessed how pairing during semantic training influenced the acquisition of perceptual expertise in the two training groups. RT and d′ were analyzed in a Pairing (Congruent/Incongruent) × Session (post-semantic/post-individuation) × Object type (Symmetric-organic/Asymmetric-metallic: each category was paired with either animate or inanimate attributes) × Categorization level (Basic/Subordinate) ANOVA, respectively. RT and d' results (Figures 5A,B) revealed different aspects of conceptual influences: RT showed a long-lasting pairing effect throughout the tests, whereas d' showed an effect of conceptual association type only after semantic training.

In RT, object matching was faster after individuation training than after semantic training, *F*_(1, 22)_ = 41.17, ${\tilde{\eta }}_{p}^{2}$ = 0.65, *p* < 0.0001. The basic-level advantage was present, *F*_(1, 22)_ = 109.6, ${\tilde{\eta }}_{p}^{2}$ = 0.83, *p* < 0.0001, with faster recognition at the basic level compared to the subordinate level. The basic-level advantage was smaller for Symmetric-organic Greebles than Asymmetric-metallic Greebles, *F*_(1, 22)_ = 12.82, ${\tilde{\eta }}_{p}^{2}$ = 0.37, *p* = 0.002. Critically, the Congruent pairing group showed a reduced basic-level advantage compared to the Reversed pairing group, as revealed by an interaction between Pairing and Categorization level, *F*_(1, 22)_ = 7.12, ${\tilde{\eta }}_{p}^{2}$ = 0.24, *p* = 0.014. The interaction of Pairing, Category level, and Session was not significant, *F*_(1, 22)_ = 0.11, ${\tilde{\eta }}_{p}^{2}$ = 0.005, *p* = 0.74, nor was any other effect (*p*s > 0.31). Thus, visual-conceptual *pairing* impacted both matching performance and a marker of perceptual expertise: associations with congruent conceptual facilitated perceptual judgments, relative to incongruent associations.

The basic-level advantage was also present in d', *F*_(1, 22)_ = 75.35, ${\tilde{\eta }}_{p}^{2}$ = 0.77, *p* < 0.0001. All other results were not significant (all *p* > 0.09) except for an unexpected result regarding the *type* of conceptual associations. This was a 4-way interaction of Group, Session, Object type and Categorization level, *F*_(1, 22)_ = 6.69, ${\tilde{\eta }}_{p}^{2}$ = 0.23, *p* = 0.017. Although a 4-way interaction could be difficult to interpret, the result essentially revealed that immediately after semantic training, both groups showed a smaller basic-level advantage for Greeble categories associated with inanimate attributes compared with the categories associated with animate attributes (*p*s < 0.006). However, following individuation training the basic-level advantage no longer differed depending on animate or inanimate associations (*p*s > 0.32). Unlike the effect of visual-conceptual *pairing* in RT that was observed both after semantic and individuation training, the *type* of conceptual associations had an initial impact on matching, but the effect was absent once the conceptual associations were no longer emphasized.

## Discussion

We found an implicit bias to relate animate concepts to unfamiliar symmetric, animal-like objects, and to relate inanimate concepts to unfamiliar asymmetric, tool-like objects. This is a rare and important experimental demonstration that the processing of novel objects is far from neutral conceptually. Moreover, whether visual and conceptual information are associated in a congruent or incongruent manner influences visual processing of untrained objects from the category. These effects last long after associations are no longer task-relevant.

Consistent with previous work, we found that concepts can be quickly associated with novel objects (Dixon et al., 1997; Gauthier et al., 2003; James and Gauthier, 2003, 2004), and that learning distinctive semantic associations can facilitate subordinate-level processing (Gauthier et al., 2003). Our results also led us to speculate that such conceptual associations, especially right after they were freshly learned, may in some tasks block the automatic activation of semantic information evoked by the visual features themselves. This conjecture is based on the absence of an interaction between semantics and visual appearance in the word judgment task only in Session 2.

For the first time we considered the effect of different kinds of pairings of conceptual information with novel objects, information that was either congruent or incongruent with the animacy of the visual appearance. We found that both congruent and incongruent pairings of objects and concepts can be learned. Moreover, these associations generalize to an object category, as they influenced performance for untrained objects during a visual matching task.

Specifically, congruent visual-conceptual pairings facilitated the acquisition of subordinate-level perceptual expertise, resulting in a smaller basic-level advantage in the Congruent than Incongruent pairing group. When learning to individuate objects, observers not only utilize visual information, they are affected by conceptual cues implied from visual features. The new associations introduced during semantic training interacted with the initial conceptual biases for the objects, such that congruent cues from different sources facilitate forming precise representations for visually similar exemplars in the trained categories.

On the other hand, the fact that even relatively unexpected conceptual associations (e.g., inanimate attributes to animal-like objects) generalized to objects that shared only some of the visual properties of the trained objects suggests a mechanism to explain the implicit bias observed in the word judgment task for novel objects prior to any training. We showed that unfamiliar objects from a novel category (e.g., symmetric-organic objects) appear to derive conceptual meaning on the basis of visual similarity with familiar categories (e.g., animals or people). Likewise, unfamiliar objects from recently familiarized categories (i.e., the untrained objects in the trained categories in the current study) derive conceptual meaning on the basis of visual similarity to objects from a recently learned category. If relatively novel and arbitrary associations that run contrary to much of our experience can generalize in this manner, a lifetime's history of conceptual learning likely has a very powerful influence on how we represent any object we encounter.

Additionally, while the main focus of the study is on the interaction between visual and conceptual properties, we found transient effects regarding the type of conceptual information on object processing immediately after associations were learned. For instance, objects associated with inanimate attributes showed less of a basic-level advantage compared to objects associated with animate attributes. One possibility is that inanimate concepts possess lower feature overlap than animate concepts (Mechelli et al., 2006). Two objects that are “elastic, shiny and antique” vs. “eco-friendly, plastic and durable” may seem to be quite different and likely to belong to different basic-level categories. Conversely, two objects that are “adorable, funny and sensitive” and “cheerful, talented and forgiving” are more likely two individuals within the same basic-level category. Therefore, inanimate associations may be more distinctive than animate associations, facilitating visual discrimination (Gauthier et al., 2003). Note, however, this difference cannot account for the pairing effect, because identical sets of associations were used for both training groups. Also, this effect regarding the type of associations faded once the associations were no longer emphasized, even though the visual-conceptual pairing effects remained. Further research should aim to replicate and explore the different temporal dynamics of the more short-lived effect of distinctive conceptual associations, and the congruency of the visual-conceptual associations, which were longer-lasting.

Several influential object recognition theories focus almost entirely on visual attributes of objects (e.g., Marr, 1982; Biederman, 1987; Perrett and Oram, 1993; Riesenhuber and Poggio, 1999; Jiang et al., 2007), assuming that conceptual associations should have no influence on object recognition (e.g., Pylyshyn, 1999; but see Goldstone and Barsalou, 1998). Additionally, researchers interested in the role of shape in object processing have often used novel objects to prevent influences from non-visual information, such as object names, familiarity and conceptual content (e.g., Op de Beeck et al., 2008). Our findings suggest that novel objects are not necessarily conceptually neutral, and that both visual and conceptual factors, and their interaction are important in the formation of object representations.

### Conflict of interest statement

The authors declare that the research was conducted in the absence of any commercial or financial relationships that could be construed as a potential conflict of interest.

## Footnotes

^1^Trials that involved the word “curvy” were discarded because of chance performance across participants.

^2^For the trained objects, the two training groups showed comparable improvement in this task after individuation training, with comparable magnitude of reduction in basic-level advantage. These results were consistent with the results from the individuation training procedures whereby both groups successfully learned to quickly and accurately identify this subset of the exemplars at the subordinate-level
