# Supplementary material for: Visual appearance interacts with conceptual knowledge in object recognition
Source: Front Psychol. 2014 Jul 29;5:793. doi: 10.3389/fpsyg.2014.00793 (PMC4114261; doi:10.3389/fpsyg.2014.00793)
Supplement: Supplementary file 1 [file DataSheet1.DOCX]

**Appendix A.** List of animate and inanimate attributes used.

| **Animate** (used in training) | **Animate** (used in word judgment task) | **Inanimate** (used in training) | **Inanimate** (used in word judgment task) |
| --- | --- | --- | --- |
| Adorable | Agreeable | Assembled | Accessible |
| Alert | Apathetic | Breakable | Affordable |
| Cheerful | Appreciative | Durable | Antique |
| Excited | Bored | Eco-friendly | Bent |
| Funny | Bossy | Elastic | Bouncy |
| Grateful | Brave | Imported | Boxy |
| Hopeful | Clumsy | Magnetic | Bumpy |
| Jealous | Confused | Matte | Compact |
| Motherly | Curious | Plastic | Cumbersome |
| Rebellious | Cute | Portable | Curvy |
| Selfish | Envious | Shiny | Geometric |
| Sensitive | Forgiving | Slippery | Expensive |
| Spontaneous | Friendly | Solid | Flimsy |
| Sweet | Gifted | Spotless | Glossy |
| Talented | Happy | Twisted | Hard-to-find |
| Thoughtful | Helpful | Uneven | Hardwearing |
| Warm | Innocent | Useful | Lopsided |
| Worried | Lonely | Well-made | Metallic |
|  | Nervous |  | Pointy |
|  | Poised |  | Prickly |
|  | Proud  Resentful  Shy  Sympathetic |  | Rubbery  Second-hand  Sparkling  Synthetic |

**Appendix B.** List of names (2-syllable nonsense words) used in the individuation training.

Aklo, Bota, Cama, Dipo, Fado, Galu, Hafi, Inra, Jepu, Kimo, Lomu, Masu, Nali, Ongi, Pila, Qifa, Raho, Subo, Tazi, Vasi, Wali, Xedo, Yuju, Zupa
